# Recurrent Upper Gastrointestinal Bleeding due to Gastric Arteriovenous Malformation

**DOI:** 10.1155/crgm/4276818

**Published:** 2026-06-29

**Authors:** Lavender Otom, Priyanka Panwar, Gerald Odong, Peter Chacha Magabe, Christopher Opio, Saleem Abdulkarim

**Affiliations:** ^1^ Department of Internal Medicine, The Aga Khan University Hospital, Nairobi, Kenya, aku.edu; ^2^ Department of Radiology, The Aga Khan University Hospital, Nairobi, Kenya, aku.edu; ^3^ Department of Radiology, Interventional Radiology Unit, University of Nairobi, Nairobi, Kenya, uonbi.ac.ke

## Abstract

We present the case of a 45‐year‐old male with repeated episodes of upper gastrointestinal bleeding due to a gastric arteriovenous malformation (AVM). The case underscores the diagnostic challenges, multidisciplinary decision‐making, and management considerations associated with gastrointestinal vascular malformations. Given the rarity of gastric AVMs and their potential for serious complications, this report highlights the importance of an integrated approach to diagnosis and treatment, including advanced imaging and endovascular interventions.

## 1. Introduction

Upper gastrointestinal bleeding (UGIB) remains a significant cause of morbidity and mortality, with peptic ulcer disease being the most common etiology [1, 2]. Although rare, arteriovenous malformations (AVMs) can also be a cause of UGIB and may occasionally result in life‐threatening hemorrhage [3]. Gastric AVMs account for approximately 1.4% of all intestinal AVMs and 1%‐2% of UGIB cases [4, 5].

A true AVM is a congenital vascular anomaly characterized by abnormal direct connections between arterial and venous structures through an intervening nidus, resulting from incomplete differentiation of the embryonic vascular plexus [5, 6]. These lesions consist of clusters of arterial, capillary, and venous vessels within the submucosa [4]. Congenital AVMs are more common in younger individuals and may occur in atypical locations such as the gastric, duodenal, and jejunal submucosa. In contrast, acquired AVMs are typically seen in older patients with comorbidities including cardiovascular disease, aortic stenosis, Von Willebrand disease, end‐stage kidney disease, CREST syndrome, and hereditary hemorrhagic telangiectasia [3, 7].

Due to their rarity, heterogenous clinical presentation, variable endoscopic appearance, and imaging challenges, gastric AVMs can be difficult to diagnose. We describe a 45‐year‐old male with recurrent UGIB ultimately diagnosed with a vascular malformation in the gastric fundus. This case highlights the need for high index of suspicion, utilizing advanced diagnostic modalities, and adopting a multidisciplinary approach to management.

## 2. Case Presentation

### 2.1. History and Physical Examination

A 45‐year‐old male with no significant past medical history was referred to our institution following two episodes of UGI bleeding. The first episode was characterized by epigastric pain followed by large‐volume coffee‐ground emesis. The second episode was more severe and was associated with syncope following vomiting, raising concern for hemodynamic instability.

The patient had a history of cigarette smoking but denied use of nonsteroidal anti‐inflammatory drugs (NSAIDs), anticoagulants, alcohol, or any history suggestive of chronic liver disease. He also denied recurrent epistaxis and had no personal or family history of bleeding disorders.

On examination, he appeared acutely ill, diaphoretic, and markedly pale, with cool extremities. There was no jaundice, pedal edema, oral or palmar telangiectasia, or other stigmata of chronic liver disease. His blood pressure was 95/59 mmHg and heart rate 103 bpm. Abdominal examination demonstrated diffuse tenderness with guarding, but no palpable masses. A hemic murmur was noted on cardiovascular examination.

### 2.2. Investigations

A complete blood count demonstrated normocytic anemia, with a hemoglobin level of 6.9 g/dL and a mean corpuscular volume (MCV) of 88.2 fL. White blood cell count (7.97 × 10^9^/L) and a platelet count (210 × 10^9^/L) were within normal limits. Liver function tests were unremarkable: total bilirubin 4.0 μmol/L, AST 17.7 U/L, ALT 7.87 U/L, ALP 41.8 U/L albumin 33 g/L, and INR 1.24. Liver stiffness measurement was 3.80 kPa, and abdominal ultrasound demonstrated a normal liver with no evidence of portal hypertension.

Twelve hours after admission, following initial fluid resuscitation and transfusion of one unit of packed red blood cells, an esophagogastroduodenoscopy (EGD) was performed. The EGD revealed prominent fundic gastric mucosal folds with two submucosal nodular lesions in the gastric fundus with overlying mucosal erythema suggestive of recent bleeding (Figure 1). The pulsatile nature of the lesions raised suspicion for an underlying vascular abnormality including AVM, Dieulafoy lesion, or isolated fundal gastric varices. Given that no active bleeding was observed during the procedure, and considering the submucosal nature of the AVM, no active endoscopic intervention was undertaken. There were no esophageal varices or endoscopic features of portal hypertension and the remainder of the stomach and duodenum appeared normal.

**FIGURE 1:**
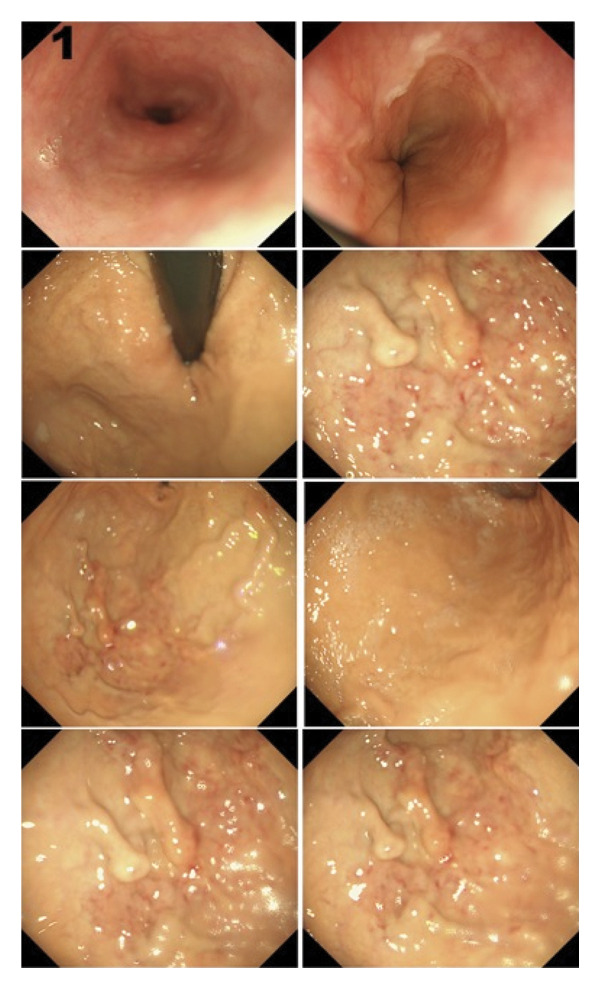
Endoscopic images at admission show prominent fundal gastric mucosal folds with linear nodular lesions in the gastric fundus. These were observed to be pulsatile and thought to represent an underlying vascular malformation. No esophageal varices were identified.

Given the endoscopic findings and the unavailability of endoscopic ultrasound in our facility, contrast‐enhanced computed tomography (CT) of the abdomen was performed to further characterize the lesions and guide management. CT demonstrated a tangle of prominent tortuous arteries within the submucosa at the gastric fundus, supplied by the short gastric arteries, branches of the splenic artery, and the left gastric artery, in keeping with a gastric vascular malformation (Figure 2). No aneurysm was identified. Venous drainage occurred through a tributary of the short gastric vein into the splenic vein. Hyperdense gastric luminal contents adjacent to the vascular malformation, measuring approximately 65 Hounsfield units (HU), were seen and were consistent with hematoma from recent hemorrhage. There was no contrast extravasation to suggest active bleeding, and no imaging evidence of portal hypertension.

**FIGURE 2:**
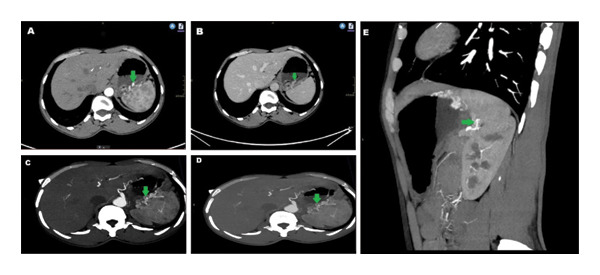
CT scan findings: (A) axial view of the arterial phase showing the tangle of prominent tortuous arteries within the submucosa (green arrow) at the gastric fundus supplied by the short gastric arteries, branches of the splenic artery and left gastric artery. No aneurysm noted; (B) venous drainage is through a tributary of the short gastric vein to the splenic vein; (C, D) axial MIP (maximum intensity projection) window showing the AVM; (E) sagittal MIP arterial view showing the AVM.

In the context of recurrent intermittent large‐volume hematemesis, the absence of traditional risk factors for UGI bleeding and imaging findings described above, a working diagnosis of gastric AVM was made.

### 2.3. Course and Management

During hospitalization, the patient experienced recurrent episodes of UGI bleeding reflected by a decline in hemoglobin from 8.1 g/dL to 6.5 g/dL, necessitating transfusion of an additional three units of packed red blood cells.

On the fourth day of admission, a multidisciplinary team meeting involving gastroenterology, surgery, and interventional radiology consultants was convened to discuss management options. The following therapeutic approaches were considered:

1. Partial gastrectomy: Although potentially definitive, surgical option was not favored because of its invasiveness, potential impact of on quality of life, and patient’s preference for less invasive treatment.
2. Celiac angiography and embolization of the vascular malformation: This was considered the preferred first‐line treatment approach.
3. Balloon‐occluded retrograde transvenous obliteration (BRTO): This option was deemed unsuitable because the lesion was not related to portal hypertension or gastric varices.

After detailed discussion with the patient and his family, the decision was made to proceed with celiac angiography and embolization of the AVM. The procedure was performed on Day 6 of admission after further hemodynamic stabilization, at which point the patient’s hemoglobin had improved to 10.2 g/dL.

Under fluoroscopic guidance, right common femoral artery access was obtained and baseline celiac digital subtraction angiography (DSA) performed. Angiography confirmed the presence of a gastric fundal AVM. Superselective cannulation of the left gastric artery demonstrated that the AVM was supplied entirely by a superomedial branch of the left gastric artery and contained a large nidus (Figure 3). Using a microcatheter, the feeding vessel was selectively catheterized, and embolization of the AVM successfully performed using Onyx, a liquid embolic agent (Figure 3D). Postembolization angiography demonstrated complete obliteration of the AVM and feeding artery.

**FIGURE 3:**
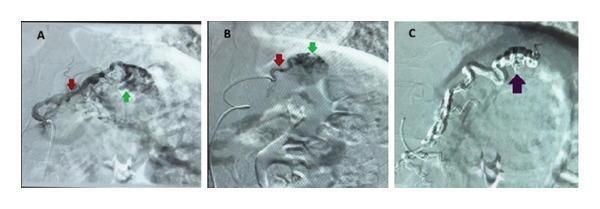
Embolization of the gastric AVM. (A and B) Note is made of the arterial supply of the AVM by a hypertrophied left gastric artery (red arrow). (C) Onyx, a liquid embolic agent was sequentially injected into the AVM (green arrow) via a microcatheter, filling the nidus and main trunk of the LGA, completely obliterating the AVM (purple arrow).

The patient remained hemodynamically stable following the procedure and was discharged 3 days later. Follow‐up evaluations at 2, 3, 6, and 12 months revealed no recurrence of GI bleeding.

## 3. Discussion

A key highlight of our case is the presentation of a relatively young patient without conventional risk factors for UGIB who developed acute massive hemorrhage with recurrent bleeding. Such a presentation should raise suspicion for an underlying vascular malformation [8]. In the gastrointestinal tract, AVMs typically present with bleeding due to the abnormal arterial‐venous connections that expose the venous system to abnormally high pressures [9]. Gastric AVMs can present across a broad clinical spectrum, ranging from asymptomatic disease and chronic occult bleeding with melena and iron deficiency anemia to acute massive hemorrhage with hemodynamic instability and recurrent bleeding, as seen in this case [9–12]. Some studies report that patients with colonic AVMs experience an average of four episodes of rectal bleeding before a diagnosis is established [3].

In this case, following hemodynamic stabilization, EGD was prioritized for both diagnostic and therapeutic purposes, in keeping with the standard of care of UGIB [13]. The finding of a pulsatile submucosal nodular lesion raised suspicion for several differential diagnosis in the context of the patient’s clinical presentation. Such a finding may be characteristic of Dieulafoy lesions, isolated gastric varices, and more rarely gastric AVMs. Nonvascular submucosal lesions arising from the mesenchyme or adjacent submucosal tissue structures were also considered in the differential diagnosis, including gastrointestinal stromal tumors and neuroendocrine tumors among others, particularly where bleeding may occur secondary to ulceration of the overlying mucosa. Given the absence of active bleeding during endoscopy and the unavailability of EUS to further characterize the lesion, contrast‐enhanced CT of the abdomen represented the most appropriate next diagnostic modality available in our setting.

Despite the widespread use of endoscopy in the evaluation of UGIB, there are no pathognomonic endoscopic findings that definitively establish the diagnosis of gastric AVMs. In an effort to standardize endoscopic characterization, Moreto et al. proposed a classification system for gastric and duodenal AVMs based on a series of 47 cases [3]. The authors described three distinct endoscopic patterns: bright red lesions with fond‐like margins, telangiectatic lesions, and submucosal nodular lesions, the latter being the pattern observed in our case [3]. According to Moreto et al., the submucosal nodular subtype posed the greatest diagnostic and therapeutic challenge likely because of its resemblance to other vascular and submucosal lesions [3]. Although this classification provides a useful framework for describing AVMs, it may not encompass the full spectrum of endoscopic appearances, highlighting the need for more comprehensive reporting systems [14, 15].

Mesenteric angiography remains the gold standard for the diagnosis of gastrointestinal AVMs [8]. Angiographic findings may include direct evidence of bleeding, such as contrast extravasation, as well as indirect features including a cluster of vessels, a hypervascular mass, arteriovenous fistulous communication, or an enlarged feeding artery supplying abnormal vascular tufts with early persistent filling of a dilated draining vein [7, 8, 14]. Selective angiography is valuable in delineating the extent and vascular anatomy of the malformation and can aid in planning definitive therapy [6]. The reported diagnostic accuracy of angiography ranges from 50%–75%, increasing to nearly 90% when performed during active bleeding [16].

EUS also plays an important complementary role in the evaluation of submucosal AVMs. In addition to characterizing the lesion, EUS with Doppler imaging can assess vascular flow patterns and help distinguish AVMs from other submucosal or vascular lesions, thereby complementing conventional endoscopic and angiographic findings [10, 11]. On EUS, AVMs typically appear as hypoechoic structures within the submucosal layer, although lesions may still be missed in up to 22% of cases [13].

In patients who undergo surgical resection, histopathological examination remains important for definitive confirmation of the diagnosis and assessment of complete excision. This is clinically relevant because recurrent gastrointestinal bleeding has been reported following incomplete resection of AVMs [3, 8, 13].

A Dieulafoy lesion was a major differential consideration in this case based on both the clinical presentation and endoscopic findings. Dieulafoy lesions are characterized by the presence of a large, persistent, tortuous submucosal artery that can result in significant bleeding through a minute mucosal defect or even intact‐appearing mucosa, with otherwise normal surrounding mucosa [17–19]. They mostly occur in the stomach, with majority located within 6–10 cm of the gastroesophageal junction along the lesser curvature [18]. Endoscopically, visualization of a pulsatile vessel with or without active bleeding through normal‐appearing mucosa is a well‐described feature and closely resembled the findings in our patient, making distinction on endoscopic grounds alone difficult [18].

However, the findings on contrast‐enhanced CT of the abdomen and DSA were more consistent with a gastric AVM and helped establish the diagnosis. Angiography in Dieulafoy lesions typically demonstrate a tortuous ectatic artery or active contrast extravasation arising from an otherwise normal‐appearing vessel [18]. EUS, if available, may also aid differentiation, as Dieulafoy lesions are characteristically seen as tortuous submucosal vessels adjacent to a mucosal defect [18, 20].

Isolated cardio‐fundal gastric varices were also considered in the differential diagnosis. However, the absence of underlying chronic liver disease, splenic vein thrombosis, or pancreatic pathology, together with the radiologic findings, made this diagnosis unlikely. Imaging studies also ruled out other submucosal lesions including GISTs.

Management of gastrointestinal AVMs is individualized according to the severity of bleeding, hemodynamic status, lesion characteristics, and locally available expertise. Therapeutic decision making is further influenced by the size, number, and anatomic location of the lesions [3, 12, 21].

The choice of interventional radiology‐based technique in this case reflects the advances in diagnosis and management of AVMs with strategies shifting to endoscopic and minimally invasive techniques depending on the presenting lesion. Selective angiography not only delineates the vascular anatomy and extent of malformation but facilitates targeted embolization to achieve hemostasis while minimizing ischemic complications. Embolization may be performed using coils and liquid embolic agents, with successful treatment depending largely on accurate identification and obliteration of the nidus, the core component of the AVM responsible for persistent shunting and recurrent bleeding [15, 22]. The role of multidisciplinary team discussion while taking the patient’s wishes into consideration is vital in these cases. In Kenya, very few centers offer interventional radiology services, and this might limit its utility to referral centers where this service is offered.

Alternative minimally invasive techniques have emerged. BRTO, originally developed for the management of gastric varices, has been successfully adopted in selected cases of gastric AVMs, particularly following failed embolization, providing an effective alternative to surgery [22, 23]. Following endovascular intervention, EUS may be useful in confirming complete obliteration of the lesion and assessing for residual vascular flow [6].

Endoscopic therapies including endoclip placement may provide control for luminal bleeding especially in localized lesions [6, 10]. Despite advances in minimally invasive therapy, surgical interventions remain important in cases where endoscopic or radiologic management is unsuccessful, technically unfeasible or when definitive resection is required [9, 10]. Histopathologic examination following resection also provides definitive confirmation of the diagnosis and assessment of complete excision. This is particularly relevant given the recognized risk of recurrent bleeding after incomplete treatment. In a review by Moreto et al., 37% of patients who underwent surgical excision experienced recurrent bleeding, attributable to incomplete resection, initial misdiagnosis, or the later development of AVMs elsewhere in the gastrointestinal tract. Notably, lesions that were not completely eradicated rebled in all reported cases [3].

The diagnostic challenges posed by gastric AVM are further illustrated by a case reported by Rios et al., involving a cirrhotic patient with recurrent UGIB initially presumed to be variceal in origin. The patient underwent multiple transjugular intrahepatic portosystemic shunt (TIPS) procedures before the true etiology, a gastric AVM, was identified and successfully treated with partial gastrectomy [9]. This case underscores the importance of maintaining a high index of suspicion for AVMs in patients with recurrent unexplained gastrointestinal bleeding.

## 4. Conclusion

Gastric AVMs are a rare but important cause of recurrent UGIB and may present significant diagnostic challenges because of their variable endoscopic appearance and resemblance of other vascular submucosal lesions. The case highlights the importance of maintaining a high index of suspicion for vascular malformations in patients with recurrent bleeding and atypical endoscopic findings. It also demonstrates the value of multimodal imaging, particularly selective angiography, in both confirming the diagnosis and facilitating minimally invasive therapy through embolization. A multidisciplinary approach remains essential to optimize management and guide contingency planning in complex cases.

### 4.1. Limitation

A 12‐month postprocedural follow‐up period may be insufficient to draw definite conclusions regarding the long‐term success and durability of the interventional radiology procedure.

## Author Contributions

Lavender Otom and Priyanka Panwar contributed to the writing and preparation of the manuscript. Gerald Odong provided the radiological images and their interpretation. Peter Chacha Magabe and Saleem Abdulkarim were involved in the clinical management and care of the patient. Christopher Opio and Saleem Abdulkarim supervised the writing, review, and finalization of the manuscript.

## Funding

No funding was received for the preparation of this manuscript.

## Disclosure

All authors reviewed and approved the final version of the manuscript.

## Ethics Statement

The patient allowed personal data processing, and written informed consent for publication was obtained from the patient, and ethical approval for publication was granted by the Institution’s Ethics and Research Committee.

## Consent

Please see the Ethics Statement.

## Conflicts of Interest

The authors declare no conflicts of interest.

## Data Availability

All data relevant to this case report have been included within the manuscript. No additional data are available for review.
